# Single mean arterial blood pressure drops during stroke thrombectomy under general anaesthesia are associated with poor outcome

**DOI:** 10.1007/s00415-020-09701-x

**Published:** 2020-01-18

**Authors:** Simon Fandler-Höfler, Stefan Heschl, Placido Argüelles-Delgado, Markus Kneihsl, Eva Hassler, Marton Magyar, Andreas Kainz, Andrea Berghold, Kurt Niederkorn, Hannes Deutschmann, Franz Fazekas, Thomas Gattringer

**Affiliations:** 1 Department of Neurology, Medical University of Graz, Auenbruggerplatz 22, 8036 Graz, Austria; 2 Department of Anaesthesiology and Intensive Care Medicine, Medical University of Graz, Graz, Austria; 3 Division of Neuroradiology, Vascular and Interventional Radiology, Medical University of Graz, Graz, Austria; 4 Institute for Medical Informatics, Statistics and Documentation, Medical University of Graz, Graz, Austria

**Keywords:** Stroke, Large vessel occlusion, Thrombectomy, Blood pressure, Anaesthesia, Neurocritical care

## Abstract

**Background:**

We examined the influence of periprocedural blood pressure (BP), especially critical BP drops, on 3-month functional outcome in stroke patients undergoing mechanical thrombectomy (MT) under general anaesthesia (GA).

**Methods:**

We screened all patients with anterior circulation large vessel occlusion receiving MT under GA at our centre from January 2011 to June 2016 and selected those who had continuous invasive periinterventional BP monitoring. Clinical and radiological data were prospectively collected as part of an ongoing cohort study, monitoring data were extracted from electronic anaesthesia records. We used uni- and multivariable regression to investigate the association of BP values with unfavourable outcome, defined as modified Rankin Scale scores 3–6 3 months post-stroke.

**Results:**

115 patients were included in this study (mean age 65.3 ± 13.0 years, 55.7% male). Periinterventional systolic, diastolic, and mean arterial BP (MAP) values averaged across MT had no effect on outcome. However, single BP drops were related to unfavourable outcome, with absolute MAP drops showing the highest association compared to both systolic and relative BP drops (with reference to pre-interventional values). The BP value with the strongest association with unfavourable outcome was identified as an MAP ever < 60 mmHg (*p* = 0.01) with a pronounced effect in patients with poor collaterals. An MAP < 60 mmHg remained independently associated with poor functional outcome in multivariable analysis (*p* < 0.01).

**Conclusions:**

For patients undergoing MT under GA, single MAP drops < 60 mmHg are independently related to unfavourable 3-month outcome. Therefore, every effort should be made to prevent periinterventional hypotensive episodes, especially below this threshold.

**Electronic supplementary material:**

The online version of this article (10.1007/s00415-020-09701-x) contains supplementary material, which is available to authorized users.

## Introduction

Mechanical thrombectomy (MT) has become the recommended treatment for acute ischemic stroke caused by anterior circulation large vessel occlusion (LVO) [1]. While there is no debate regarding a higher chance for improved outcome with successful recanalization, it is not yet clear if MT should be performed under general anaesthesia (GA), conscious sedation (CS) or local anaesthesia alone. Recent single-centre randomized controlled trials found no difference in outcome between these approaches [2–4], while larger observational studies suggested a predominantly worse outcome for thrombectomy patients under GA [5, 6].

One reason for this could be the greater challenge of blood pressure management during the induction of GA where post-induction hypotension is common due to cardio-depressive and vasodilatory side effects of most anaesthetic agents [7]. There is, however, conflicting evidence whether hypotension during MT is independently related to worse outcome [8–12]. Previous studies on the relation of blood pressure (BP) during MT and outcome also vary in their suggestions of BP target levels and whether absolute BP levels or relative changes in BP from pre-intervention levels are more critical [8, 10]. The 2018 AHA/ASA stroke guidelines note that BP should be kept ≤ 180/105 mmHg during MT and recommend correction of hypotension and hypovolemia but do not indicate what minimal BP should be maintained [13]. Anaesthesiologic guidelines recommend preservation of a systolic BP > 140 mmHg during MT, but this is not based on hard data [14]. It is also unclear whether systolic, diastolic or mean arterial blood pressure (SAP/DAP/MAP) has the greatest impact on outcome in the context of MT. Additionally, the influence of single critical BP drops during MT has not been investigated in depth and no specific cut-off values have been discovered yet. A commonly recommended minimal threshold for adequate cerebral blood flow autoregulation in the absence of increased intracranial pressure is a mean arterial pressure (MAP) ≥ 60 mmHg [15]. Moreover, BP drops are likely particularly deleterious in patients with poor collaterals, but this has not been specifically investigated in MT patients thus far [16].

In view of these uncertainties, we explored the impact of different BP values (absolute and relative BP changes, SAP, DAP, and MAP) and their course during MT on outcome. Specifically, we hypothesized that critical BP drops of MAP ≤ 60 mmHg during MT were related to worse 3-month functional outcome. For this purpose, we analysed consecutive anterior LVO stroke patients who underwent GA for MT and had continuous invasive BP monitoring.

## Methods

### Study participants

For this retrospective cohort study, we identified all patients ≥ 18 years who had received MT because of anterior circulation LVO stroke (i.e. occlusion of the intracranial internal carotid artery and/or middle cerebral artery in the M1 or M2 segments) between January 2011 and June 2016.

**Fig. 1:**
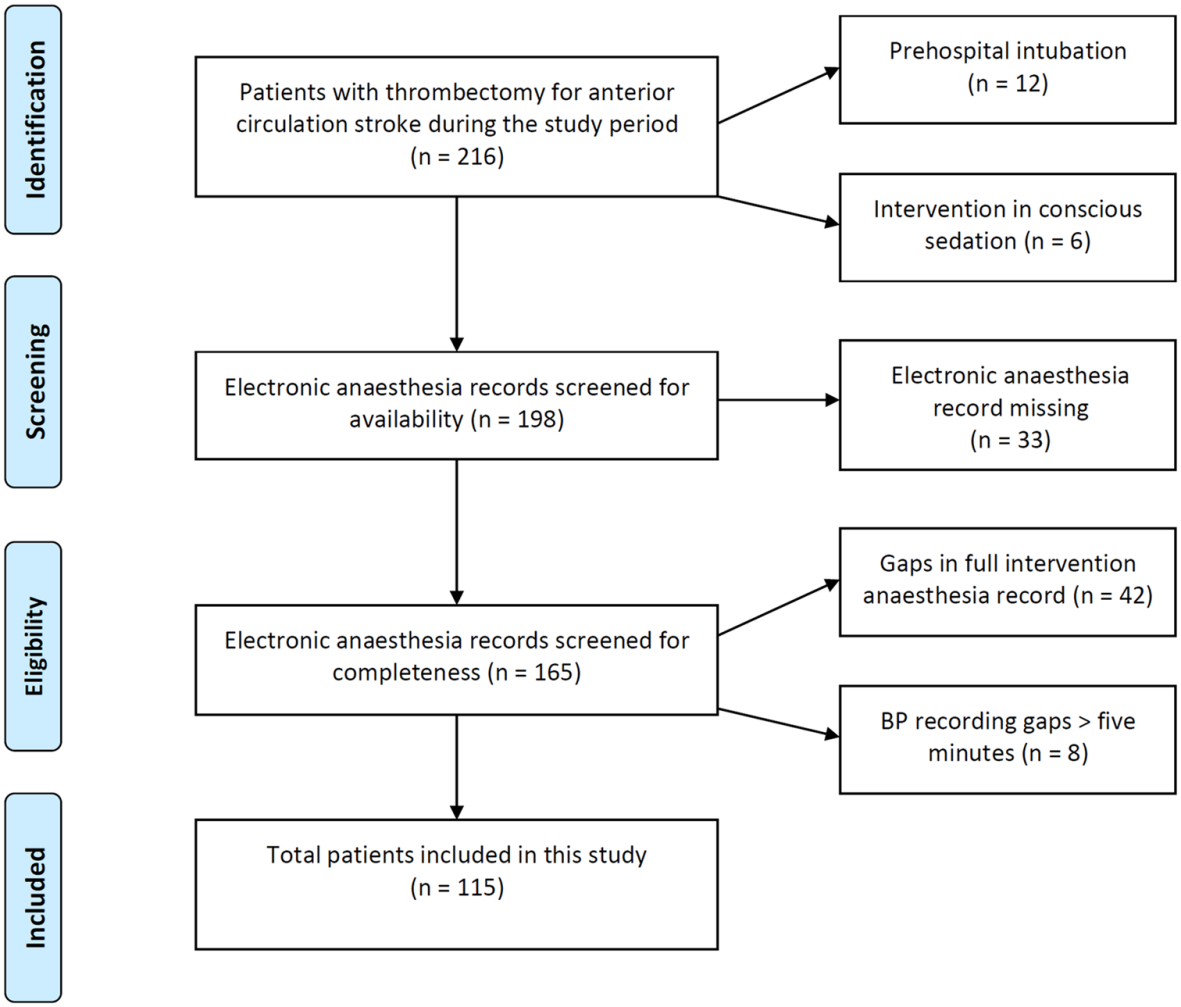
Study flowchart

In our centre, MT is routinely performed by interventional radiologists using stent retrievers under GA by neuroanaesthesiologists. Type and doses of anaesthetic and blood pressure modifying drugs were individually chosen by the treating neuroanaesthesiologist. As we aimed for a uniform and well-characterized study cohort, patients were excluded from this study if they were intubated prehospitally or in the referring hospital or if they were not intubated at all during thrombectomy. Also, patients were excluded if electronic anaesthesia records were incomplete (BP measurement gaps of  ≥ 5 min). Paper-only anaesthesia records were excluded, as we aimed to only include exact and complete hemodynamic documentations. A flowchart of patient selection can be found in Fig. 1.

### Data assessment

**Fig. 2:**
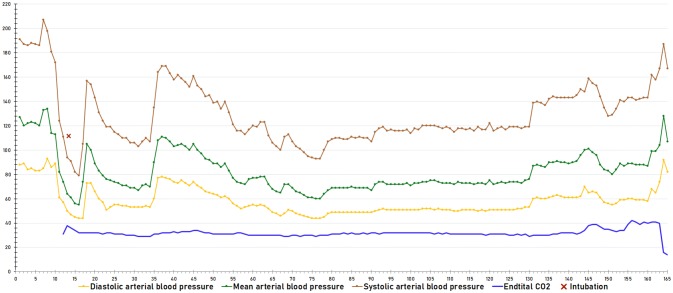
Continuous electronic periinterventional blood pressure monitoring in a representative patient, showing a severe blood pressure drop after induction of anaesthesia (red cross)

Clinical and radiological data were prospectively collected as part of an ongoing cohort study of all patients undergoing thrombectomy at our centre. Pre- and postinterventional Alberta stroke program early CT scores (ASPECTS) as well as collateral scoring on initial CT angiography using the TAN scale [17] were performed by two experienced neuroradiologists blinded to clinical data. MT was defined as successful if postinterventional Thrombolysis in cerebral infarction (TICI) scales of 2b or more were achieved. Periinterventional vital signs (heart rate; systolic, diastolic and mean arterial BP and end-expiratory CO_2_, measured every minute) and anaesthesia management were extracted from electronic anaesthesia records, encompassing the time period from patient arrival in until departure from the angio suite (example shown in Fig. 2). BP measurement was done using invasive BP monitoring via peripheral arterial lines in all cases.

The primary outcome variable was functional neurological outcome at 3 months according to the modified Rankin Scale (mRS) and dichotomized as good (scores of 0–2) or unfavourable (scores 3–6). Furthermore, ordinal mRS shift analysis was performed. mRS scores were rated by stroke physicians during a scheduled visit at the stroke outpatient department.

### Statistical analysis

Statistical analysis was performed using IBM SPSS Statistics 22. Normally distributed continuous variables were compared by the unpaired Student’s *t* test, for other distributions the Mann–Whitney *U* test was used. For categorical variables, we used the Chi-square test and univariable logistic regression. *p* values of less than 0.05 were considered statistically significant. We calculated receiver operating characteristic (ROC) curves for analysis of sensitivity and specificity of BP values towards functional outcome. All variables with *p* < 0.1 in the univariable analysis were entered in a binary multivariable regression model.

The study was approved by the ethics committee of the Medical University of Graz. Anonymized datasets generated during this study are available from the corresponding author upon reasonable request.

## Results

### Patient characteristics and outcome

From 216 patients who received MT for anterior LVO during the study period, 115 were included in this study. Patients had to be excluded because MT had been performed under conscious sedation (*n* = 6), intubation had been performed before arriving at our angio suite (*n* = 12), electronic anaesthesia records were not available (*n* = 33) or because of measurement recording gaps (*n* = 50; Fig. 1).

Their age ranged from 27 to 85 years (mean 65.3 ± 13.0 years) and 55.7% were men. There were no differences in clinical or radiological findings between included and excluded patients aside from slightly higher NIHSS scores in the latter (Table 1).

**Table 1:** Clinical and radiological data of the study cohort regarding study inclusion and 3-month outcome

	Study cohort	Excluded patients	*p* value	Study cohort		
				mRS 0–2	mRS 3–6	*p* value
	*n* = 115	*n* = 101		*n* = 59 (51.3%)	*n* = 56 (48.7%)	
Clinical data						
Age (mean ± SD)	65.3 ± 13.0	66.7 ± 13.9	0.43	62.1 ± 13.8	68.7 ± 11.1	0.001
Male sex	64 (55.7%)	51 (50.5%)	0.53	31 (52.5%)	33 (58.9%)	0.49
Hypertension	72 (62.6%)	67 (66.3%)	0.66	29 (49.2%)	43 (76.8%)	0.002
Dyslipidaemia	17 (14.8%)	20 (19.8%)	0.33	7 (11.9%)	10 (17.9%)	0.37
Chronic heart disease^a^	17 (14.8%)	17 (16.8%)	0.55	5 (8.5%)	12 (21.4%)	0.05
Diabetes	16 (13.9%)	23 (22.8%)	0.06	3 (5.1%)	13 (23.2%)	0.005
Atrial fibrillation	48 (41.7%)	40 (39.6%)	0.85	22 (37.3%)	26 (46.4%)	0.32
Prestroke mRS (median, IQR)	0 (0)	0 (0)	0.47	0 (0)	0 (0)	0.13
Stroke of unknown symptom onset	27 (23.5%)	13 (12.9%)	0.05	11 (18.6%)	16 (28.6%)	0.21
NIHSS at admission (median, range)	14 (4–25)	15 (6–34)	0.01	13 (4–22)	16 (8–25)	< 0.001
Radiological findings						
MCA/M1-occlusion	87 (75.7%)	76 (75.2%)	0.95	44 (74.6%)	43 (76.8%)	0.78
MCA/M2-occlusion	13 (11.3%)	4 (4.0%)	0.05	7 (11.9%)	6 (10.7%)	0.85
Intracranial ICA occlusion	11 (9.6%)	18 (17.8%)	0.08	5 (8.5%)	6 (10.7%)	0.68
CTA collateral scoring (TAN, median, range)	2 (0–3)	2 (0–3)	0.16	2 (0–3)	1 (0–3)	0.04
ASPECTS pre-intervention (median, range)	9 (5–10)	9 (3–10)	0.20	9 (5–10)	9 (3–10)	0.05
Intervention						
Intravenous thrombolysis	69 (60%)	68 (67.3%)	0.43	39 (66.1%)	30 (53.6%)	0.17
Time to groin puncture (min, median, IQR)	200, 82	205, 84	0.56	203, 81	197, 74	0.55
Time to recanalization (min, median, IQR)	260, 93	271, 90	0.36	254, 92	272, 77	0.68
Anaesthesia duration (min, median, IQR)	112, 64	NA	NA	108, 54	131, 67	0.02
Successful recanalization (TICI 2b–3)	96 (83.5%)	82 (81.2%)	0.43	56 (94.9%)	40 (71.4%)	0.001
ASPECTS post-intervention^b^ (median, range)	5 (0–9)	5 (0–9)	0.41	6 (2–9)	3 (0–8)	< 0.001

*ASPECTS* Alberta stroke program early CT scores, *CTA* computed tomography angiography, *ICA* internal carotid artery, *MCA* middle cerebral artery, *NIHSS* National Institutes of Health Stroke Severity Scale, *mRS* modified Rankin Scale, *TICI* thrombolysis in cerebral infarction

^a^Coronary artery disease, heart failure, cardiomyopathy or valve disease

^b^At follow-up imaging 24 h post-stroke

Most patients were treated for an occlusion of the M1-segment of the middle cerebral artery (75.7%). Sixty percent received intravenous thrombolysis prior to MT. Median NIHSS at admission was 14, median pre-interventional ASPECTS was 9, successful recanalization (TICI 2b–3) was achieved in 83.5% of patients.

About half of the patients (51.3%) had favourable 3-month functional outcome (mRS 0–2) and 14 patients (12.2%) had died. Patients with unfavourable outcome (mRS 3–6) were older, had more severe stroke syndromes according to the NIHSS and more often had hypertension and diabetes. Worse collaterals on admission CT angiography and unsuccessful recanalization as well as lower pre- and especially postinterventional ASPECTS were also associated with poor 3-month outcome (Table 1).

### Periinterventional blood pressure

Periinterventional BP drops were frequent. On average, the lowest periprocedural BP values recorded were 87, 46 and 60 mmHg (SAP/DAP/MAP). When averaging BP values over the entire recording period, there was an average drop of about 40% compared to preinterventional values and single values were often much lower (Table 2).

**Table 2:** Blood pressure values and frequency of drops in MAP < 60 mmHg related to 3-month outcome

	mRS 0–2	mRS 3–6	*p* value
	*n* = 59 (51.3%)	*n* = 56 (48.7%)	
Preinterventional SAP (mean ± SD)	148.8 ± 26.4	151.9 ± 26.5	0.54
Preinterventional MAP (mean ± SD)	105.9 ± 19.7	107.2 ± 20.7	0.73
Preinterventional DAP (mean ± SD)	80.4 ± 14.0	81.3 ± 16.4	0.75
Average SAP (mean ± SD)	124.3 ± 17.2	122.6 ± 17.4	0.60
Average MAP (mean ± SD)	82.2 ± 12.1	78.6 ± 15.5	0.17
Average DAP (mean ± SD)	61.3 ± 9.9	58.8 ± 8.5	0.16
Minimal SAP (mean ± SD)	88.9 ± 22.0	85.6 ± 18.6	0.39
Minimal MAP (mean ± SD)	61.7 ± 12.2	58.3 ± 11.6	0.13
Minimal DAP (mean ± SD)	47.5 ± 9.6	44.8 ± 8.3	0.10
Lowest relative SAP^a^ (mean ± SD)	58.2% ± 16.3	61.2% ± 16.2	0.32
Lowest relative MAP^a^ (mean ± SD)	56.2% ± 14.6	60.0% ± 15.0	0.16
Lowest relative DAP^a^ (mean ± SD)	57.2% ± 14.8	60.5% ± 14.5	0.22
Patients with MAP ever below 60 mmHg	27 (45.8%)	39 (69.6%)	0.01

*DAP* diastolic blood pressure, *MAP* mean arterial pressure, *SAP* systolic blood pressure

^a^Relative to preinterventional values

**Fig. 3:**
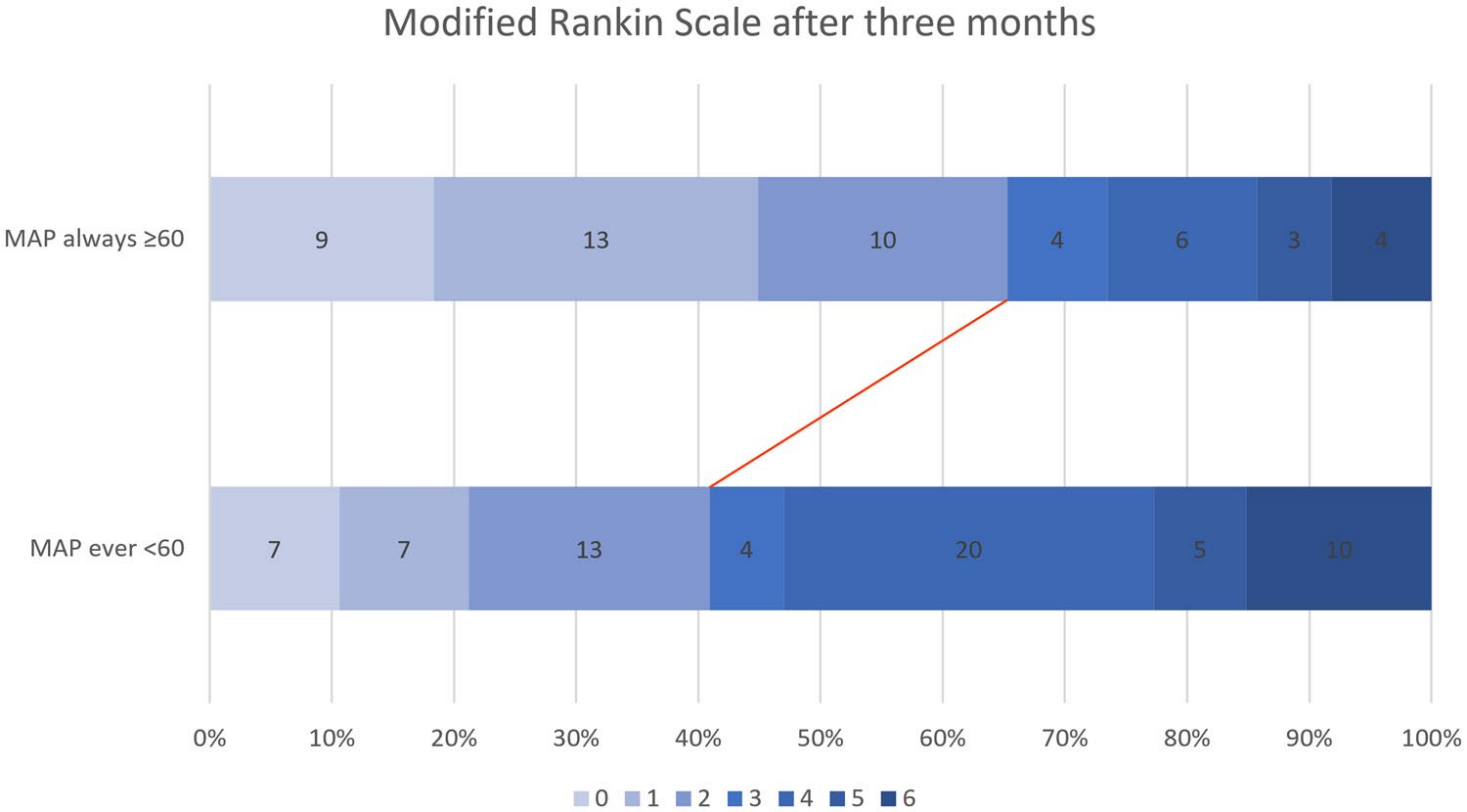
Modified Rankin Scale scores at 3 months of patients without and with periinterventional MAP drops below 60 mmHg, showing worse functional outcome of patients with such drops (*p* < 0.01)

In univariable analysis, preinterventional, average procedural and minimal BP values were not associated with 3-month outcome. However, a periinterventional MAP that fell below 60 mmHg at any time was associated with worse outcome (mRS 3–6 at 3 months post-stroke, *p* = 0.01, OR 2.72, 95% CI 1.26–5.85, Table 2). Ordinal mRS shift analysis at 3 months also showed worse outcome for patients with an MAP drop < 60 mmHg (*p* < 0.01, Fig. 3). There were no differences in demographic, clinical or radiological findings between patients whose periinterventional MAP dropped below 60 mmHg or not (Table 3).

**Table 3:** Comparison of patients who had an MAP drop < 60 mmHg during intervention or not

	MAP < 60 mmHg	MAP ≥ 60 mmHg	*p* value
	*n* = 66 (57.4%)	*n* = 49 (42.6%)	
Clinical data			
Age (mean ± SD)	66.3 ± 12.6	64.0 ± 13.5	0.36
Male sex	37 (56.1%)	27 (55.1%)	0.92
Hypertension	45 (68.2%)	27 (55.1%)	0.15
Dyslipidaemia	13 (19.7%)	4 (8.2%)	0.09
Chronic heart disease^b^	8 (12.1%)	9 (18.4%)	0.35
Diabetes	10 (15.2%)	6 (12.2%)	0.66
Atrial fibrillation	26 (39.4%)	22 (44.9%)	0.55
Stroke of unknown symptom onset	16 (24.2%)	11 (22.4%)	0.82
NIHSS at admission (median, range)	14 (4–25)	15 (5–23)	0.82
Radiological findings			
MCA/M1-occlusion	49 (74.2%)	38 (77.6%)	0.68
MCA/M2-occlusion	6 (9.1%)	7 (14.3%)	0.38
Intracranial ICA occlusion	7 (10.6%)	4 (8.2%)	0.66
CTA collateral scoring (TAN, median, range)	2 (0–3)	2 (0–3)	0.26
ASPECTS pre-intervention (median, range)	9 (5–10)	9 (3–10)	0.45
Intervention			
Intravenous thrombolysis	36 (54.5%)	33 (67.3%)	0.17
Time to groin puncture (min, median, IQR)	208, 85	196, 85	0.19
Time to recanalization (min, median, IQR)	265, 81	245, 91	0.32
Anaesthesia duration (min, median, IQR)	120, 58	106, 57	0.12
Successful recanalization (TICI 2b–3)	54 (81.8%)	41 (83.7%)	0.86
Post-intervention			
mRS 3 months post-stroke (median, range)	4 (0–6)	2 (0–6)	< 0.01
Mortality at 3 months post-stroke	10 (15.2%)	4 (8.2%)	0.26
Periinterventional blood pressure			
Preinterventional SAP (mean ± SD)	150.4 ± 28.7	154.1 ± 26.1	0.49
Preinterventional MAP (mean ± SD)	105.5 ± 21.9	109.6 ± 16.2	0.30
Preinterventional DAP (mean ± SD)	80.4 ± 16.6	81.4 ± 13.2	0.73
Minimal SAP (mean ± SD)	77.9 ± 20.0	100.0 ± 12.5	< 0.001
Minimal MAP (mean ± SD)	53.6 ± 10.4	68.9 ± 7.6	< 0.001
Minimal DAP (mean ± SD)	42.1 ± 7.7	51.6 ± 7.8	< 0.001
Average SAP (mean ± SD)	118.2 ± 17.0	130.6 ± 15.0	< 0.001
Average MAP (mean ± SD)	76.3 ± 10.8	86.0 ± 15.8	< 0.001
Average DAP (mean ± SD)	56.5 ± 8.6	65.0 ± 7.9	< 0.001
Lowest relative SAP^a^ (mean ± SD)	54.0% ± 16.4%	67.7% ± 12.5%	< 0.001
Lowest relative MAP^a^ (mean ± SD)	52.7% ± 14.4%	65.8% ± 11.9%	< 0.001
Lowest relative DAP^a^ (mean ± SD)	54.2% ± 13.3	65.1% ± 14.2	< 0.001

*ASPECTS* Alberta stroke program early CT scores, *CTA* computed tomography angiography, *DAP* diastolic blood pressure, *ICA* internal carotid artery, *IQR* interquartile range, *MAP* mean arterial pressure, *MCA* middle cerebral artery, *NIHSS* National Institutes of Health Stroke Severity Scale, *mRS* modified Rankin Scale, *SAP* systolic arterial pressure, *TICI* thrombolysis in cerebral infarction

^a^Relative to preinterventional values

^b^Coronary artery disease, heart failure, cardiomyopathy or valve disease

Using ROC curves to further investigate associations between BP values and functional neurological outcome, we found that absolute BP values were more relevant than relative values and that MAP showed a stronger association than SAP or DAP. In ROC curve analysis of absolute MAP drops, the strongest predictive value was confirmed as an MAP < 59.5 mmHg at any time during MT (sensitivity 0.60, specificity 0.63, Youden’s Index 0.23, positive predictive value 0.60, negative predictive value 0.63, supplementary Fig. 1).

In patients with poor collaterals (TAN scale score 0–1, 45.2% of patients), a periinterventional MAP below 60 mmHg was even stronger associated with unfavourable 3-month outcome (OR 4.29, 95% CI 1.12–16.39, *p* = 0.03) compared to patients with TAN scale scores 2–3 (OR 1.88, 95% CI 0.61–5.81, *p* = 0.28). Different lengths of hypotensive periods (both calculated as a continuous variable as well as dichotomized to ≥ 10 min compared to < 10 min) were not related to clinical outcome.

### Multivariable analysis

In multivariable analysis comprising all variables with a *p* value of < 0.1 in univariable analysis, an MAP drop below 60 mmHg remained an independent predictor for poor functional outcome at 3 months post-stroke (OR 6.17, 95% CI 1.57–24.36, *p* < 0.01), together with age, diabetes, chronic heart disease, NIHSS at admission, preinterventional ASPECTS and unsuccessful recanalization (detailed data in Table 4).

**Table 4:** Binary multivariable logistic regression analysis regarding poor outcome (mRS 3–6)

Test variable	Odds ratio	95% confidence interval	*p* value
Clinical data			
Age^a^	1.07	1.01–1.13	0.03
Hypertension	1.02	0.25–4.26	0.98
Diabetes	16.3	1.17–225.9	0.04
Chronic heart disease^b^	9.29	1.31–65.96	0.03
NIHSS at admission^a^	1.27	1.07–1.49	< 0.01
Radiological findings and intervention			
ASPECTS pre-intervention^a^	0.55	0.34–0.90	0.02
CTA collateral scoring (TAN)^a^	1.25	0.52–3.01	0.61
Duration of anaesthesia	0.99	0.98–1.01	0.41
Unsuccessful recanalization (TICI 0–2a)	23.2	2.17–247.9	< 0.01
Periinterventional blood pressure drop			
MAP ever below 60 mmHg	6.17	1.57–24.36	< 0.01

*ASPECTS* Alberta stroke program early CT scores, *MAP* mean arterial pressure, *mRS* modified Rankin Scale, *NIHSS* National Institutes of Health Stroke Severity Scale

^a^Per year/min/point on respective scale

^b^Coronary artery disease, heart failure, cardiomyopathy or valve disease

## Discussion

In this retrospective cohort study, we found that MAP drops below 60 mmHg during GA for MT were independently associated with worse functional outcome at 3 months post-stroke. As ideal BP targets or thresholds for patients undergoing MT have not yet been agreed upon, our work adds several aspects to this debate.

The first important point is that our results suggest that absolute BP values have a stronger impact on neurological outcome than relative changes, both during induction of anaesthesia and throughout the whole thrombectomy procedure. Cerebral autoregulation helps to provide adequate blood flow to the brain across a range of BP values, but might be impaired in pathological conditions such as stroke [18, 19]. Our results show the strongest association between unfavourable outcome and an MAP that dropped below 60 mmHg at any point in time during MT. This could indicate the lower end of the autoregulatory capacity of the brain where cerebral blood flow becomes directly dependent on BP. It needs to be considered however, that the individual autoregulatory curve of patients might well be unpredictably shifted to the right, in particular in patients with long standing arterial hypertension and also differ between different cerebral pathologies [19].

The second important finding is that MAP drops showed a stronger association with worse outcome than systolic BP drops. Notably, MAP thresholds are not included in current recommendations for anaesthesiologic management of endovascular treatment for acute ischemic stroke [14]. From a pathophysiological point of view, MAP is the most important and commonly used blood pressure target value regarding cerebral blood flow [20]. Our clinical results now support this important role of mean over systolic BP. For clinical management of patients with low MAP, it needs to be considered, that diastolic pressure has a higher impact than systolic pressure. Therefore, elevation of diastolic pressure is probably the therapeutic approach of choice and could be achieved via fluid administration in many cases. However, this might be challenging in the neurointervention suite before the induction of anaesthesia for MT, in particular in patients with cardiac or renal comorbidities as it can lead to fluid overload and cardiac decompensation. Especially in clinically hypovolemic patients, early initiation of fluid administration already in the emergency department could potentially mitigate these risks. Disproportionate elevation of SAP by vasoconstrictor agents to increase MAP on the other hand, might potentially impede microcirculatory cerebral blood flow [21].

The effect of a drop in MAP < 60 mmHg regarding unfavourable outcome was quite strong in our study cohort and—besides well-established prognostic factors—remained significant in multivariable analysis. Furthermore, there were no baseline differences between patients who dropped to an MAP < 60 mmHg versus those who always had an MAP above this critical threshold. The strict avoidance of an MAP < 60 mmHg should therefore be of paramount importance when GA is chosen for MT. This corresponds well with data from traumatic brain injury, showing that even a single episode of severe hypotension is associated with worse outcome [22]. While it could be assumed that longer duration of critical hypotensive periods leads to accumulated impact on patient outcome, we did not find such an effect. However, many hypotensive episodes in our study were short and the rather low number of longer hypotensive periods may have been too small to find statistically significant effects.

Notably, hypotensive episodes were not rare in our “real world” cohort; however, the severity and frequency of periinterventional hypotension was comparable to a previous retrospective study [8]. Post hoc analysis of the SIESTA trial in regards to BP values on the other hand revealed no association between periinterventional BP and functional outcome after 3 months [11]. However, this was a prospective trial investigating anaesthesia in MT and followed strict BP targets. Furthermore, single BP drops were not specifically explored. Therefore, the difference to our results may be explained by the different study designs and a more rigorous adherence to BP targets in the prospective study setting.

Our study may have unravelled a potential reason for the different performance of GA compared to CS for MT in small single-centre randomized controlled trials compared to observational and retrospective studies. Hypotension is more difficult to prevent in GA (especially during anaesthesia induction, after which most of the critical BP drops occurred in our study) and may occur more often (and more severe) in real-world patients compared to prospective studies which specifically investigated anaesthesia management of MT. This could be a possible—or at least partial—explanation why the prospective randomized controlled trials investigating anaesthesia for MT showed no difference in outcome between CS and GA [2–4], while observational and retrospective studies indicated worse outcomes under GA [5, 6].

Previous studies on anaesthesia management for MT have not particularly studied the pre-interventional collateralization status. Our finding that patients with worse collaterals are even more prone to worse outcome due to BP drops is in line with pathophysiological considerations [23].

This study has several strengths. All patients had invasive BP monitoring, which allowed us to precisely record BP. BP values were recorded and saved in the electronic anaesthesia records automatically. Patients with missing or incomplete records were excluded, the accuracy and data quality of BP measurements, the core variable for this study, can therefore be assumed to be very good. Notably, patients who were excluded from the study did not differ from study patients regarding important clinical baseline characteristics, except from higher NIHSS values in excluded patients, caused by the very high NIHSS values of prehospitally intubated patients (which were excluded from the study). Furthermore, clinical parameters were recorded prospectively in our study and we were able to include important radiologic parameters such as collateral status and pre- and postinterventional ASPECTS in our analysis.

Even though the retrospective nature of this study comes with known limitations, it also limits any possible observer effect in particular in regards to BP management, resulting in our study representing real-world data outside of well-controlled prospective trials on BP management in stroke. We did not explore the impact of different anaesthetic drugs and doses on both blood pressure and patient outcome as well as BP management before and after MT, which should be explored in further studies.

The uniformity of our study cohort limits the generalizability of our findings to other settings and particularly to other methods of anaesthesia (such as CS) for MT. Larger studies involving multiple centres and different anaesthetic management during MT are required to confirm our results and give further insights in periinterventional management of stroke patients.

## Electronic supplementary material

Below is the link to the electronic supplementary material.

Supplementary file1 (DOCX 478 kb)
