# Supplementary material for: Single mean arterial blood pressure drops during stroke thrombectomy under general anaesthesia are associated with poor outcome
Source: J Neurol. 2020 Jan 18;267(5):1331–9. doi: 10.1007/s00415-020-09701-x (PMC7184049; doi:10.1007/s00415-020-09701-x)
Supplement: Supplementary file 1 — Supplementary file1 (DOCX 478 kb) [file 415_2020_9701_MOESM1_ESM.docx]

**Supplementary material**


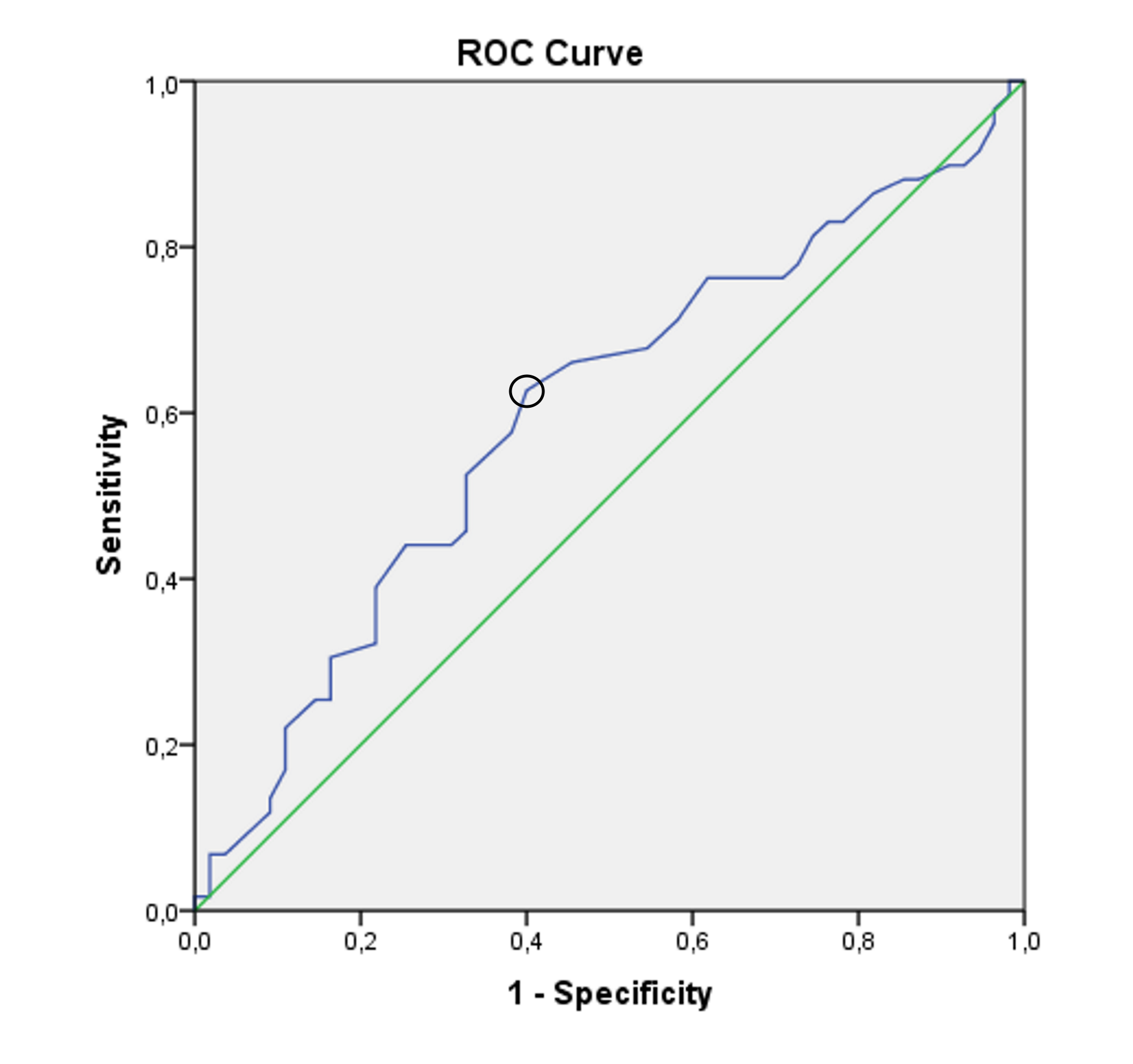


***Supplementary Fig. 1:*** *ROC (Receiver operating characteristic) curve for minimal periinterventional mean arterial blood pressure compared to unfavorable patient outcome at three months. The highest Youden’s Index is reached at 59.5 mmHg (black circle; Sensitivity 0.63, Specificity 0.60).*
